# Tattooing and immune response: A mouse model too far from clinical reality

**DOI:** 10.1073/pnas.2611211123

**Published:** 2026-07-31

**Authors:** Nicolas Kluger

**Affiliations:** ^a^ Aava Medical Center, Kerava 04200, Finland; ^b^ Department of Dermatology, Tampere University Hospital, Tampere 33520, Finland; ^c^ Tattoo Consultation, Department of Dermatology, Hopital Bichat-Claude Bernard, Paris 75018, France

The study by Capuccetti et al. on the potential impact of tattooing on vaccination response in mice (1) has attracted considerable media attention, often with an exaggerated alarmist tone. Several methodological limitations warrant discussion before any extrapolation to real-life human tattooing.

The tattoo model used is far from standard practice. The authors tattooed 25 mm^2^ of the mouse hind paw, representing approximately 30% of the entire footpad—equivalent to a fully covered 60 cm^2^ shoulder tattoo in humans (2). Such extensive single-color coverage is uncommon in practice. By contrast, Engel et al. (3) used only four single-pass stripes, a more realistic approach. It is also well established that researchers tend to deposit more ink than professional artists (4), which likely amplifies local and systemic inflammatory responses. Testing several tattoo types and densities would have strengthened the study.

Regarding vaccination timing, the two-day interval between tattooing and vaccination, while potentially of scientific interest, does not reflect real-world practice, as vaccination immediately before or after tattooing is generally discouraged (5). Of the time points studied, only the two-month results are relevant to human extrapolation.

On the findings themselves, tattooed mice produced lower COVID-19 antibody titers than nontattooed controls; however, absolute titer values would have been more informative than ratios. Reduced antibody levels do not imply absent immunity, and challenge experiments with live viruses would be needed to assess actual protection.

Finally, the divergent results between COVID-19 and influenza vaccines deserve equal attention rather than being downplayed. The authors describe tattoo ink as “altering” immune response; “modulating” would be more accurate and neutral. More broadly, one could just as well reject the possibility of vaccinating against COVID-19 within a tattoo while, conversely, proposing influenza vaccination within a tattoo—rather than selectively retaining only the former interpretation. Choosing one conclusion over the other reflects an inconsistency that undermines the overall argument.

Overall, the present study demonstrates that tattooing can modulate vaccination response in a specific mouse model that differs substantially from real-life conditions. Furthermore, the contribution of local inflammation to the observed effects remains speculative. Contrary to the authors’ assertion, no epidemiological study has to date established an association between tattoo exposure and an increased risk of lymphoma (6, 7). Finally, the authors suggest that attenuated vaccines may carry additional risk in tattooed individuals, citing the case reported by Carius et al. of military personnel who developed a localized smallpox reaction at a tattoo site—a tattoo performed shortly after vaccination. This case, however, precisely illustrates the rationale behind the established recommendation to avoid vaccination on a fresh tattoo or tattooing over a recent vaccination site (5), rather than supporting broader concern about attenuated vaccines in tattooed individuals.
